# The Kidd-Quain Embroglio

**Published:** 1881-05

**Authors:** 


THE KIDD-QUAIN EMBROGLIO.—We have waited for some time past to get at the "true inwardness" in the etiquettical commotion that has been going on among our English cousins regarding the case of the late Lord Beaconsfield, before expressing our opinion on its merits; but from recent communications which have appeared from Dr. Kidd and others, we really cannot see anything more in it than the usual "tempest in a teapot;" or, to put it in a little stronger aphorism, "Much ado about nothing." We reach this conclusion logically and easily; and such, we opine, our readers will do also, after careful perusal of Dr. Kidd's statements introduced below. If

Dr. K. speaks truthfully—and there seems to be no reason to question his veracity—*he was not* a homœopathist at all at the time of his late attendance on the British Premier, but was what the enlightened medical man of this day should be—a rational physician. As we hope to make remarks on some ethical points in a future issue of the journal, we will conclude this matter by introducing Dr. Kidd's letter. He says:

"*First.*—Although, to quote the words used, 'a reputed homœopath,' I design once for all to disclaim any such party designation. Four years ago I resigned all connection with the Homœopathic Hospital and Society. In a very extensive practice, extending over thirty-four years, I have always adopted that course of treatment which my own study and experience have taught me to be most effectual to my patients. Herein I claim to have acted as any man does who is not bound by the trammels of a merely mechanical routine. Like other practitioners, I use the drugs of the British Pharmacopœia, but in many cases I have learnt from experience that what are called 'homœopathic remedies' may be usefully prescribed. Such remedies I freely use in suitable cases, and according to my own judgment. I do not prescribe infinitesimal doses, nor according to the caprice of my patients.

"*Second.*—With regard to the co-operation of Dr. Quain with myself, the facts are as follows: A valuable life was at stake—one precious to her most Gracious Majesty the Queen, and to many millions of her subjects. I had been enabled for nearly four years to afford prompt and effectual relief to that illustrious man in many severe illnesses. From the first moment of the present attack I recognized its gravity and danger. For ten nights and days I bore the strain of incessant attention; then her Majesty wished that the responsibility of so momentous an illness should be shared in consultation. I was asked to make arrangements accordingly. Knowing the satisfaction it would give her Majesty to have Sir William Jenner's co-operation and opinion, I at once wrote to him. He replied to the following effect: 'Holding, as you and I do, different views as to practical treatment, I do not think Lord Beaconsfield's interests could in any way be served by our meeting in consultation; on the contrary, it could not be without risk to him.' Thus, without inquiring as to the manner in which I was treating the patient, Sir

William declined to meet me. Before receiving his reply, however, Lord Beaconsfield had selected Dr. Quain, who immediately communicated with me as to how I was treating the case, and upon my assurance that it was on the ordinary principles of medicine and not homœopathical, he visited the patient, thus fulfilling the spirit of that 'boast of the medical profession, that in the hour of sickness it recognizes only humanity in need of succor!' In this way Dr. Quain and I did not work together, as your contemporary supposed, without being agreed, nor did either sacrifice his convictions to effect the co-operation; on the contraıy, Dr. Quain's great skill was thus made useful to our illustrious patient, and my intimate knowledge of his constitution and of the disease was as helpful to Dr. Quain.

"*Third.*—As to the rules of professional etiquette which have been alleged herein to have been violated, it is impossible within the limits of this letter to discuss the entire question; but of this at least I am convinced, that if the boast of the medical profession above set forth is to remain a living principle of conduct, we must be guided, not by the misleading flicker of prejudice and jealousy, but rather by the clear and convincing light of humanity and common sense."

